# Revision Total Knee Arthroplasty Using Robotic Arm Technology

**DOI:** 10.1016/j.artd.2021.11.003

**Published:** 2021-12-10

**Authors:** Micah MacAskill, Baylor Blickenstaff, Alexander Caughran, Matthew Bullock

**Affiliations:** Marshall University, Joan C Edwards SOM, Department of Orthopaedics, Huntington, WV, USA

**Keywords:** Revision total knee arthroplasty, Robotic-assisted, Computer-assisted, Component survival, Revision arthroplasty

## Abstract

Total knee arthroplasty (TKA) is a highly successful operation for the treatment of end-stage osteoarthritis of the knee. Increasing use of computer-assisted and robotic-assisted total joint arthroplasty has been shown to improve component position, with short-term studies demonstrating improved survivability in unicompartmental knee arthroplasty. Robotic-assisted technology has been shown to be helpful in revising unicompartmental knee arthroplasty to TKA, as well as hip fusion to total hip arthroplasty, but few have described revision of a primary TKA. This case report describes the use of robotic-assisted technology in revision TKA. Robotic assistance during revision TKA may improve component alignment and increase prosthesis longevity. Future research is needed to investigate the effects on survivorship and cost.

## Introduction

Total knee arthroplasty (TKA) is widely regarded as one of the most successful operations for the treatment of end-stage osteoarthritis of the knee. A well-balanced and aligned TKA is capable of permitting patients to return to a high degree of function postoperatively. With recent advancements in implant technology and surgical techniques, TKA has become a viable surgical option for younger populations. As a result, the burden of total knee revision surgery is increasing [1]. Knee replacements can fail for a multitude of reasons including infection, instability, aseptic loosening, polyethylene wear, stiffness, and fracture [2]. Revision TKA is a technically challenging procedure that requires critical thinking and problem solving to achieve a satisfactory functional outcome.

In the past several years, there has been an increasing interest in computer-assisted and robotic total joint arthroplasty [3,4]. Currently, this technology is being used for unicompartmental knee arthroplasty (UKA) as well as total hip arthroplasty (THA) and TKA. Numerous studies have shown that computer-assisted technology improves component positioning and decreases “outliers,” with the theory that this will improve component longevity [[5], [6], [7], [8], [9]]. Today robotic assistance allows assessment of soft tissue balancing, which may improve patient satisfaction and outcomes, but future research is ongoing.

Traditionally, revision surgery is performed with manual instrumentation using a set of accepted “norms” (eg, distal femoral cut angle of 6 degrees, 90-degree perpendicular cut to long axis of tibia, gap balancing for assessment of femoral rotation, and so on). The surgeon relies on his or her expertise in identifying anatomic landmarks intraoperatively, like the medial femoral condyle or meniscal scar, to restore the joint line and offset and achieve ligamentous balance. Identification of these landmarks can be challenging, particularly in the setting of severe bone loss. Numerous studies have shown that restoration of the joint line in revision knee replacement is paramount in obtaining a good outcome postoperatively [10,11]. Although it is still to be determined if the improved component positioning and improved balance achieved with robotic-assisted primary TKA is clinically significant, particularly with implant longevity, its usage in revision TKA for similar purposes is appealing [3].

Surgeons continue to harness the abilities of robotics and devise ways to use new technology during surgery. There are case reports of the robot being used in the conversion of UKA to TKA, as well as conversion of previous hip fusion to THA [12,13]. Advanced imaging is frequently used for preoperative planning, which can be helpful in the restoration of native anatomy. A small series by Yun et al. in 2020 has demonstrated that using computed tomography (CT)-based robotic-assisted surgery for conversion of UKA to TKA did not result in inferior outcomes [14].

To our knowledge, there are no reports of robotic technology being used for the revision of a TKA. Computer-assisted techniques for revision TKA have been described, but as advancements are made in robotic technology, refining of revision techniques will occur [15]. We present 2 cases where the Mako robotic arm technology (Stryker, Mahwah, NJ) was used for revision TKA. Our described technique represents an off-label use of this device. Patient informed consent was obtained before this study. The use of the robotic technology and the subsequent publication was not funded by industry support.

## Case history

### Case 1

A 54-year-old female with a history of obesity, tobacco use, and osteoarthritis of the right knee underwent stage 2 replant because of history of prosthetic joint infection. The patient had initially presented for a second opinion due to continued pain after patellofemoral arthroplasty. Infection workup was positive, leading to an uncomplicated stage 1 explant with placement of a dynamic spacer. The patient cleared the infection but continued to have pain and subjective instability. She elected for stage 2 replantation TKA.

### Case 2

A 79-year-old male with a history of chronic kidney disease stage 3, type 2 diabetes mellitus, obesity, and atrial fibrillation on warfarin with a history of left TKA performed by outside physician several years prior. The patient developed symptoms of pain and instability in his left knee, which prompted a visit to our orthopedic clinic. The patient had a cruciate retaining implant with a positive posterior drawer sign and signs of mid-flexion instability. The patient initially failed conservative treatment of bracing and therapy. The patient elected for revision TKA for instability.

## Surgical technique

A traditional preoperative CT scan was obtained using the Stryker Makoplasty Protocol (Stryker, Mahwah, NJ). A Mako product specialist (MPS) was available at the time of CT scanning to ensure the quality of the scan, as patient motion and metal artifact from existing implants can degrade the imaging. The MPS then performed image segmentation and uploaded the data to the computer.

For both cases, the previous midline incision was used followed by a standard medial parapatellar arthrotomy. A synovectomy of the medial and lateral gutters as well as the suprapatellar pouch was performed to aid in exposure. Neither case required a quadriceps snip to enhance exposure, but this procedure can be performed if needed. The femoral and tibial arrays were placed in standard fashion with the femoral pins in the medial condyle away from the femoral component, and the tibial pins placed in the distal tibial diaphysis through separate stab incisions. Care must be taken in the placement of the array pins if a long stem is planned. We suggest a distance of at least 160 mm from the joint line. The femoral pins may also be placed in the femoral diaphysis through stab incisions.

**Figure 1:**
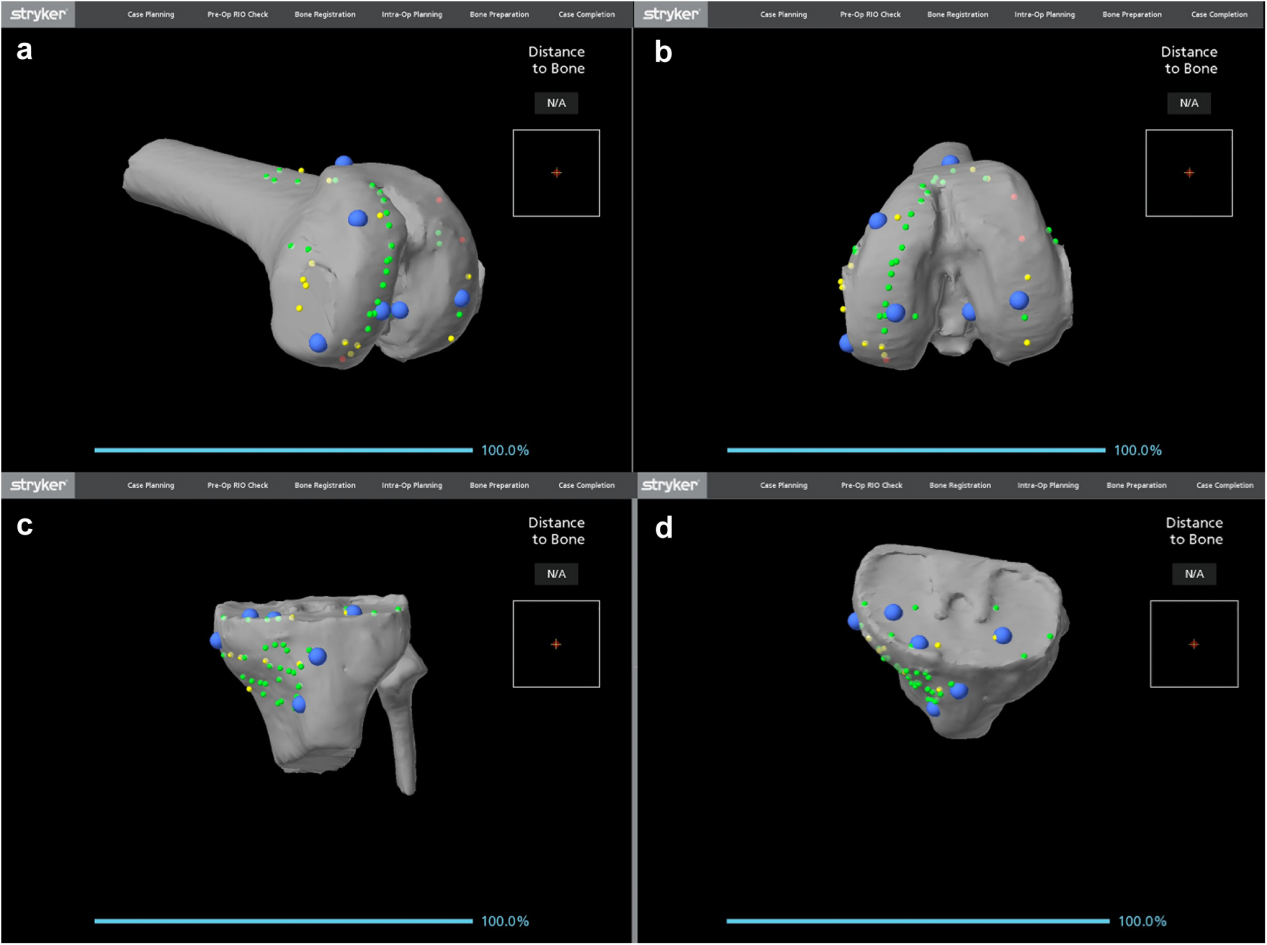
(a and b) The femoral registration used in case 2. Note that contour of the prosthesis as well as the distal femur and medial and lateral bony portions were used for highest reliability. (c and d) The tibial registration in case 2. Note in case 2, the tibial tray was still in place. The anteromedial plateau and metaphyseal bone were found to be the most reliable registration points to establish accurate registration.

Next, the polyethylene components were removed in standard fashion (For case 1, this included removal of the entire all-poly tibia.). With the existing prosthetic components in place, the extent of the components and bony anatomy of the femur and tibia were registered. Registering the anterior flange, posterior condyles, and distal extent of the femoral implant and the periphery of the tibial component as well as the medial and lateral epicondyles and anterior tibial metaphysis is performed (Fig. 1). We did not follow the proposed standard registration pattern because these points are different in a revision scenario. Registration accuracy is predicated on widespread pattern in 3-dimensional space. Owing to the presence of metal artifact on the CT scan, registration of certain points may be difficult, and therefore, additional points on bony surfaces can be obtained. Figure 1 shows the registration points used during case 2, which gave us the most accurate registration. Once registration is complete, the components were removed with standard techniques to minimize bone loss during component removal.

In case 1, the all-poly tibia was removed, while in case 2, the polyethylene insert was removed for the dynamic ligamentous stability data collection. While using the angular measurements from the computer, the extension gap was assessed with the knee held in neutral mechanical alignment while pulling longitudinal traction. For flexion, the knee was held at 90 degrees, and a laminar spreader was used for ligamentous tensioning to ensure a rectangular flexion gap. Final alterations to the component positioning were performed to achieve desired gap balancing.

**Figure 2:**
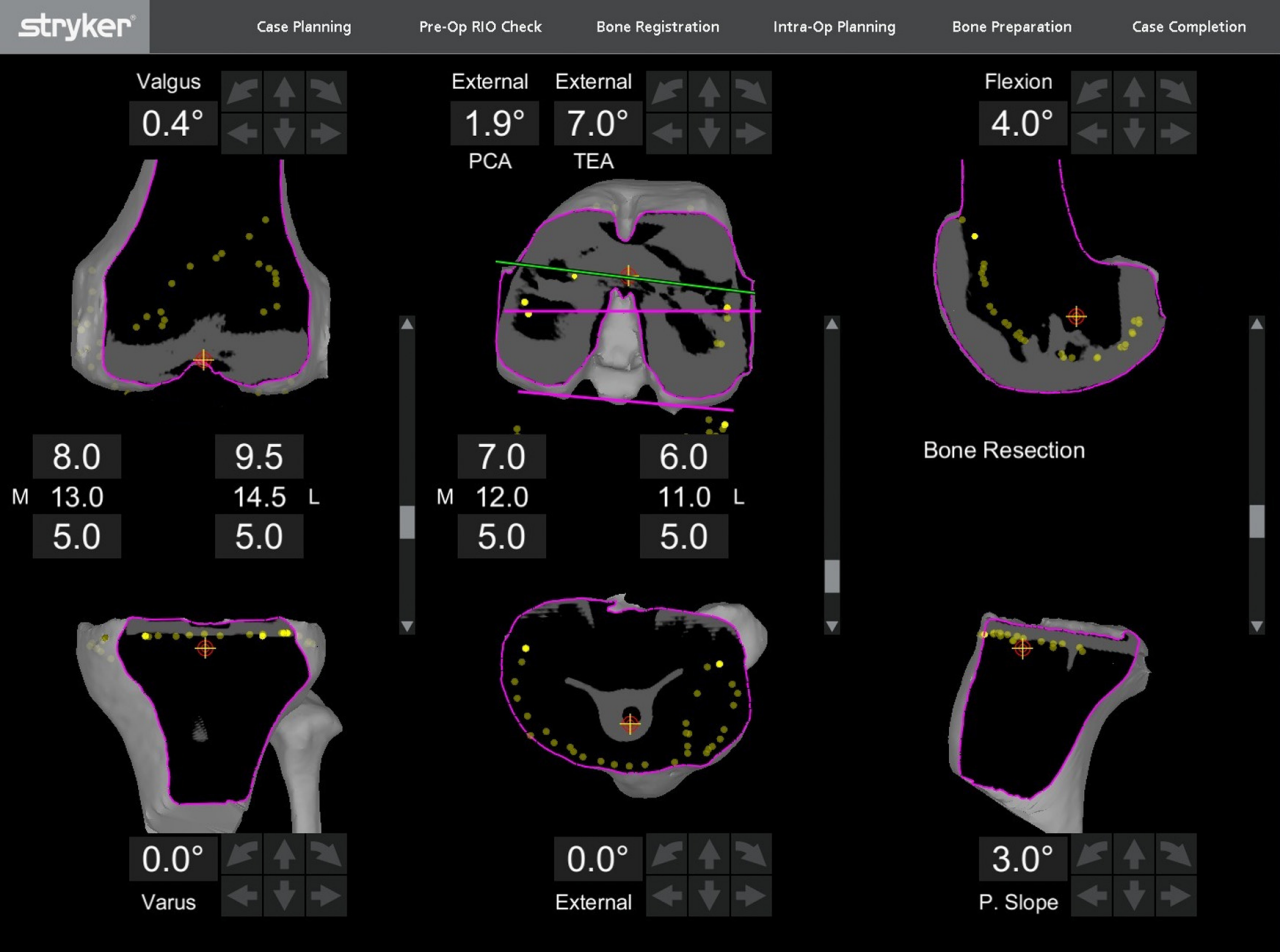
Intraoperative display demonstrating multiple registration points after component removal. The yellow dots in the figure represent the new registration points and are being shown in coronal, axial, and sagittal views using the Mako software. This information is used to aid in determination of new bony resections and the need for augments.

**Figure 3:**
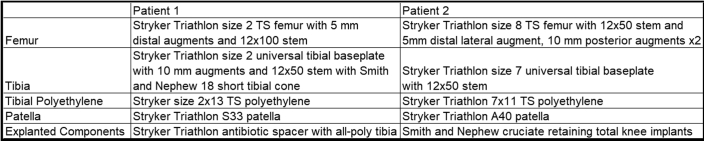
A chart of the final implants from revision surgeries of patient 1 and patient 2. Of note, patient 1 initially presented with a patellofemoral unicompartmental arthroplasty. The data listed are of stage 2 of a 2-stage revision for infection.

The green probe was then used to map the remaining bone stock of the femur and tibia to aid in planning of any augments needed as shown in Figure 2. Per surgeon preference, the bony cuts were made in the following order: distal femur, posterior chamfer, (The blade is then changed.) then posterior femur, anterior femur, anterior chamfer, and tibia. When an augment was required, the MPS moved the component appropriate amount before performing the cut. For example, when a distal femoral augment was required, the femoral component was moved proximally by 5 mm increments until reaching the appropriate level and then returned to the final position before moving to the next cut. This does require rechecking the checkpoints and sawblade with the green probe any time the component is moved. Neither case required the use of a step cut, so the authors were unable to comment on whether this technology can be used to perform one. Figure 3 shows the component sizes and augments used, and both patients received Stryker Triathlon TS components (Stryker, Mahwah, NJ). The advent of the Mako robotic arm technology allows for precise positioning of the implant (within 0.5 mm and 1 degree) to enable optimal balancing of the flexion and extension gaps.

**Figure 4:**
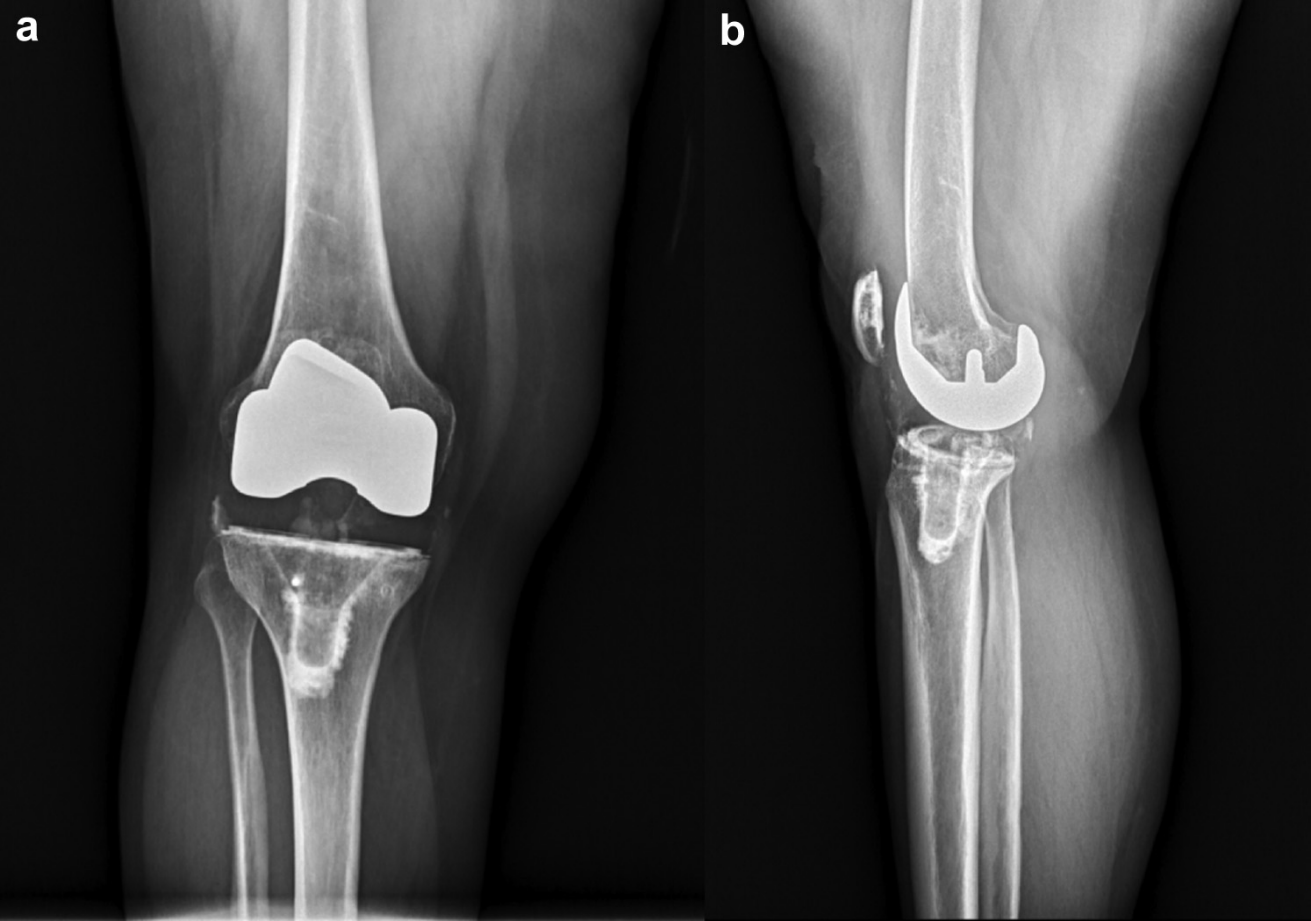
(a) Anteroposterior and (b) lateral radiographs of the right knee of case 1 demonstrate cemented right total knee arthroplasty with Stryker Triathlon cruciate retaining femur and all-polyethylene tibia in good alignment without fracture, subsidence, loosening, or osteolysis.

**Figure 5:**
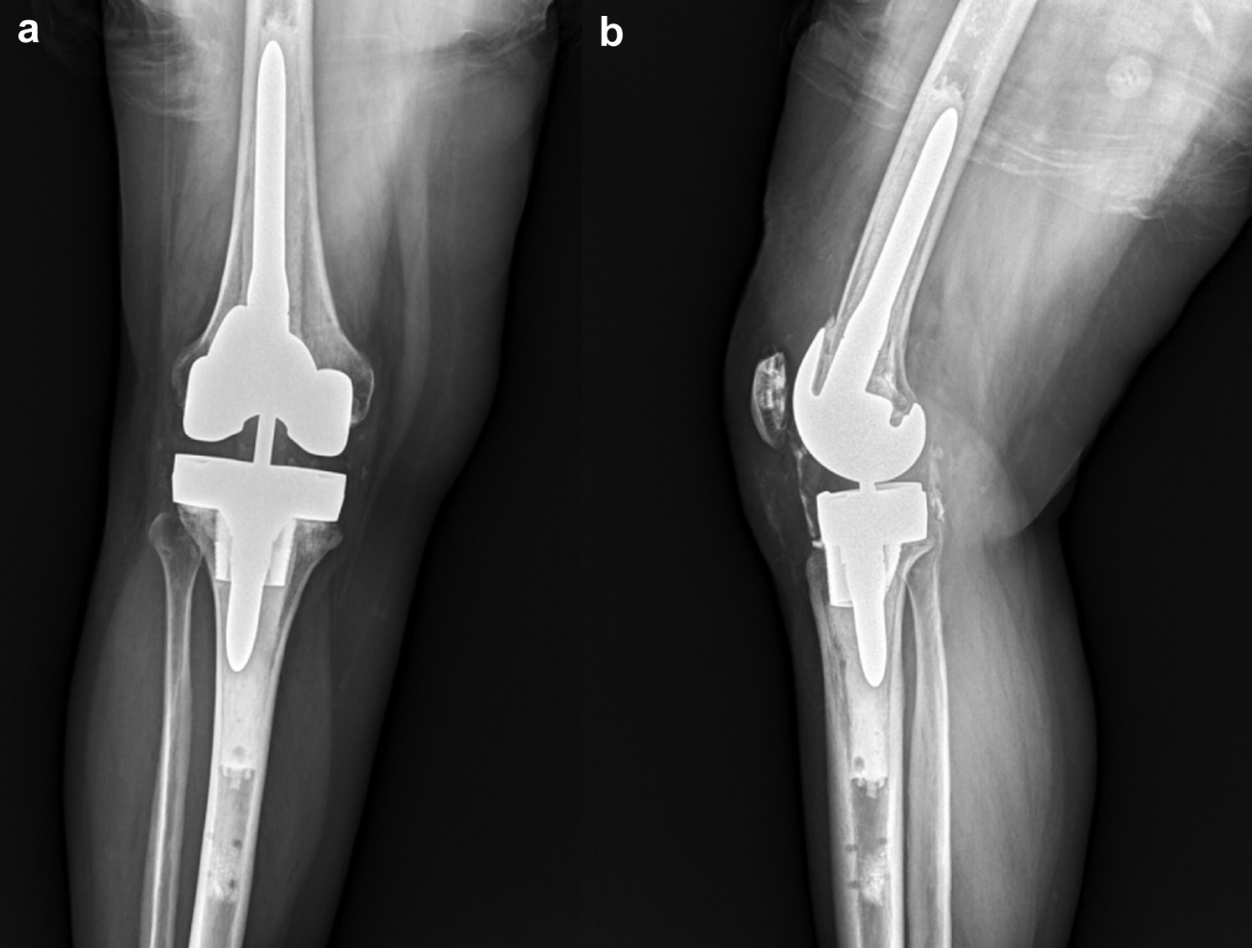
(a) Anteroposterior and (b) lateral radiographs of the right knee of case 1 demonstrate cemented revision Stryker Triathlon TS total knee arthroplasty with good alignment and no evidence of fracture, subsidence, loosening, osteolysis, or fracture about the array pin sites.

**Figure 6:**
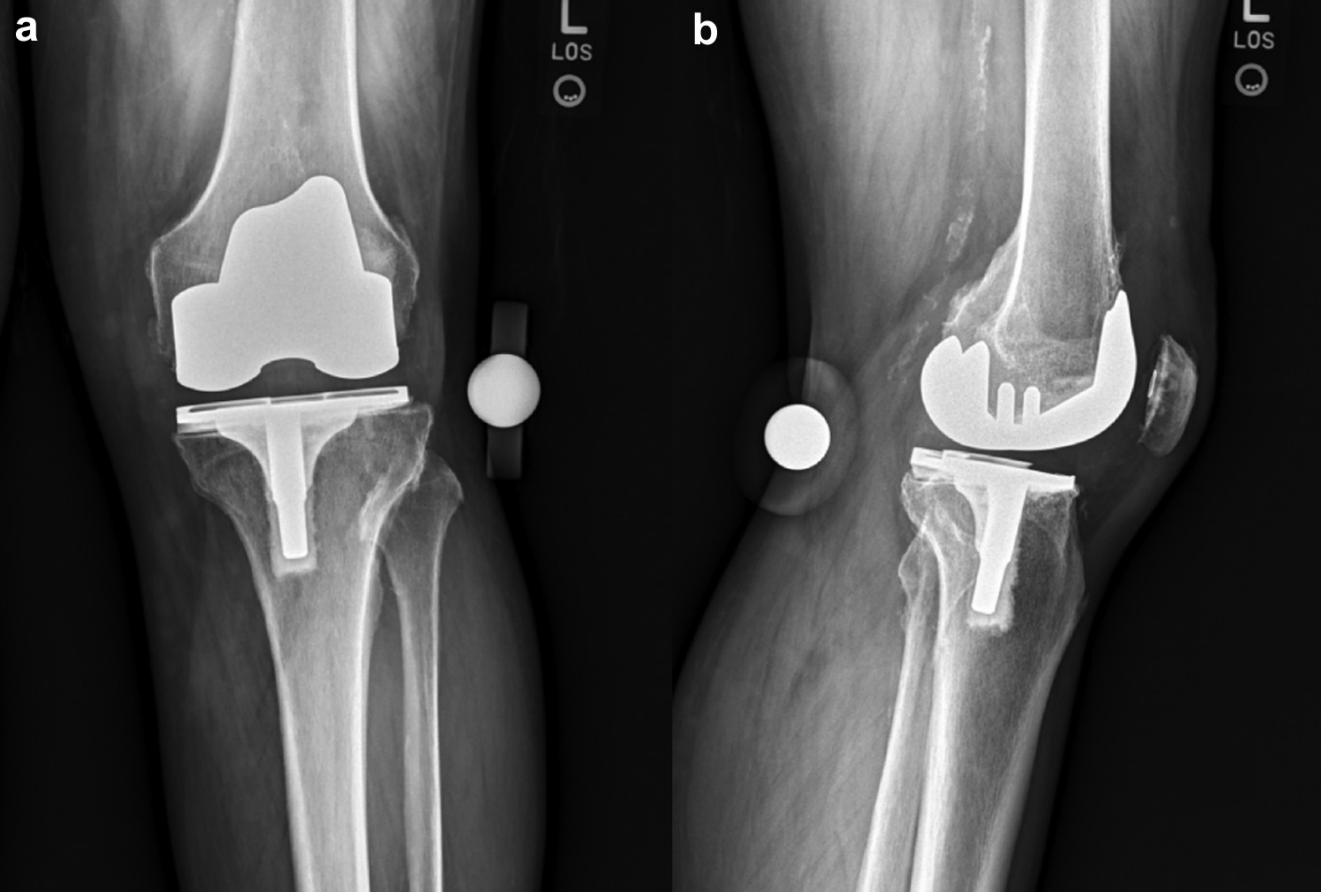
(a) Anteroposterior and (b) lateral radiographs of the left knee of case 2 demonstrate Smith and Nephew cruciate retaining design with components in reasonable alignment with lucency underneath the medial tibial baseplate. Vascular calcifications are noted. Decreased condylar offset seen on the lateral view without evidence of fracture.

**Figure 7:**
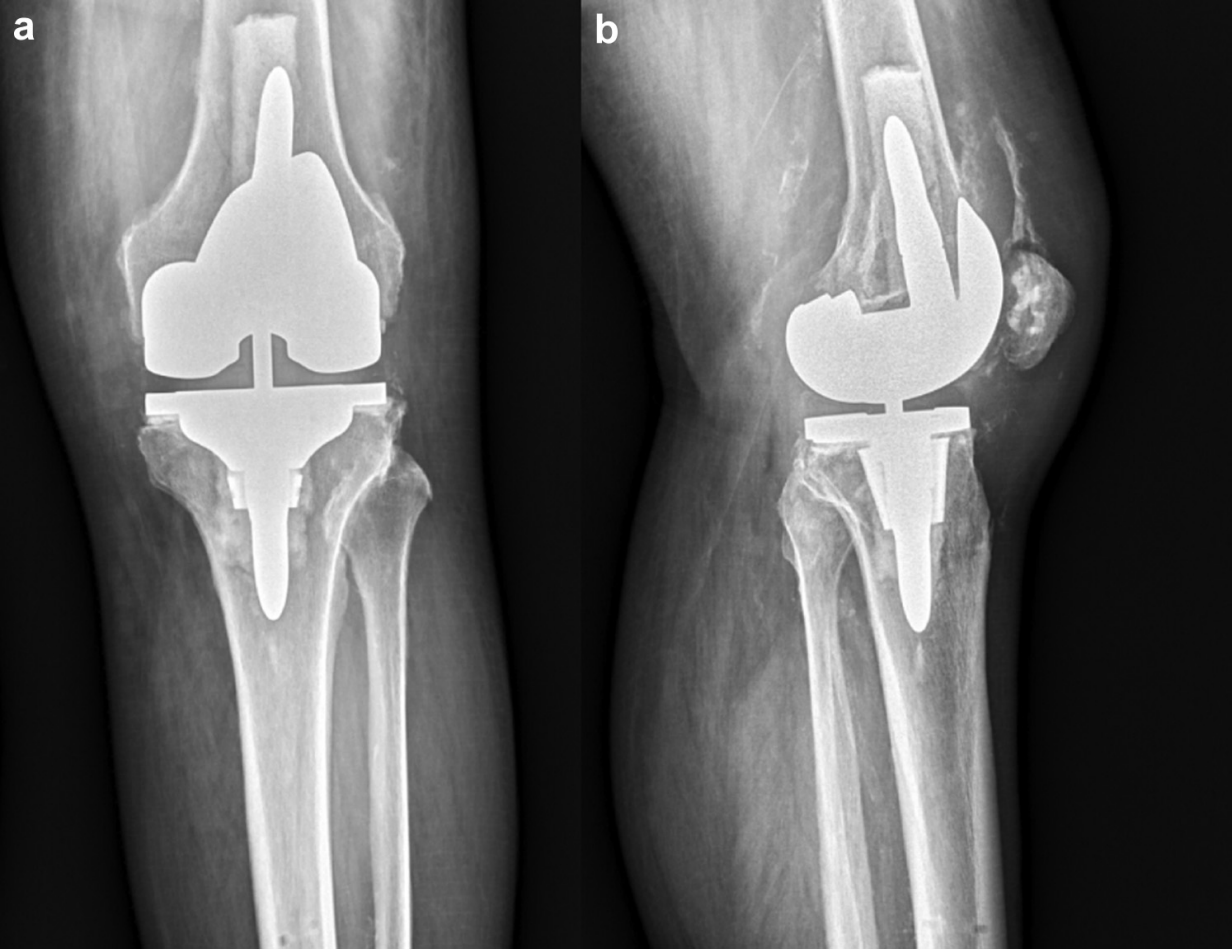
(a) Anteroposterior and (b) lateral radiographs of the left knee of case 2 demonstrate cemented revision Stryker Triathlon TS total knee arthroplasty in good alignment and no evidence of fracture, subsidence, loosening, osteolysis, or fracture about the array pin sites. Vascular calcifications are seen with the appearance of some heterotopic bone formation in the quadriceps tendon on the lateral view.

**Figure 8:**
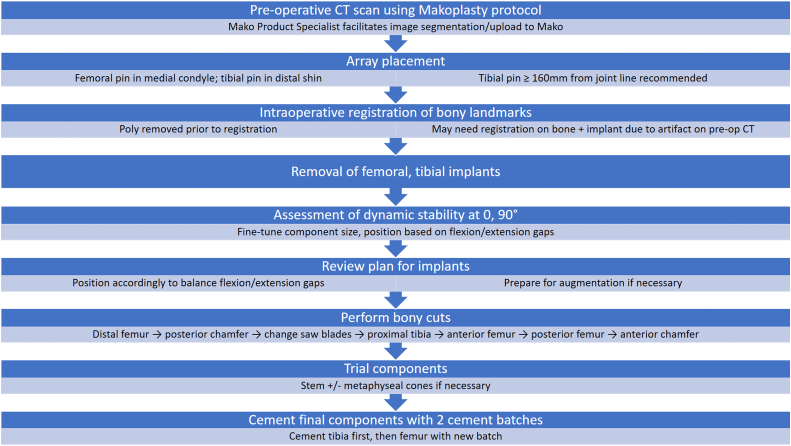
Flowchart demonstrating the workflow for Mako-assisted revision total knee arthroplasty.

The introduction of metaphyseal cones has enabled a shorter stem to be used during revision scenarios [16]. Stem and cone use was determined on a case-by-case basis depending on the bone stock available and underwent standard preparation. Cemented stem fixation was chosen in both these cases as it allowed for the femoral and tibial component alignment to be independent from the intramedullary canal. After trialing, the final components were then cemented in place in 2 separate batches with the tibial component cemented first followed by the femoral component. Canal plugs were used when needed in the femoral and tibial canals to allow for cement pressurization and to prevent egress of the cement except in case 2 where, after boss reaming, no tibial canal plug was necessary. The trial polyethylene was exchanged for the real polyethylene component after cement hardened, and the knee was closed in standard fashion. The tibial array pin sites were closed with 3-0 nylon sutures in figure-of-eight fashion. Preoperative and postoperative radiographs are shown in Figure 4, Figure 5, Figure 6, Figure 7. A workflow for performing revision Mako TKA is demonstrated in Figure 8.

## Follow-up

Patients are allowed immediate weight-bearing as tolerated with a standard TKA protocol postoperatively. Physical therapy began on postoperative day 0 once the spinal anesthesia resolved. Patients were discharged on postoperative day 1. Both patients were able to achieve full range of motion by 6 weeks postoperatively.

Patient 1 ambulated without an assistive device by 6 weeks. At her 3-month visit, she reported that her knee was much improved and felt more stable. On examination, she had 0-120 degrees of flexion at the right knee and was stable to varus and valgus stress with less than 1 mm of joint opening based on physical examination. She was doing well at 6 months postoperatively with mild soreness about the tibial array pin site with well-healed incisions and no erythema. Patient 2 had a mechanical fall at 5 weeks postoperatively and went back to using a cane. He had achieved 0-115 degrees of flexion by postoperative week 6. At his 6-month follow-up, he was ambulating without assistive devices with a normal gait despite his fall. Both recoveries were uneventful.

## Discussion

Numerous studies have shown that robotic-arm-assisted total joint replacement is more accurate in placement of components than conventional jig-based joint replacement [[17], [18], [19], [20]]. With improved accuracy in regard to restoration of joint line and balanced flexion and extension gaps, theoretically surgeons can decrease their rates of failure [21]. UKA has been showed to have better success rates at midterm follow-up when robotic-armed assistance was used [[22], [23], [24]]. In total hips, the computer-based software allows the surgeon to individualize the cup inclination/version, thereby decreasing dislocation rates [25]. Previous studies have shown that the robotic-arm assistance can be used for the conversion of UKA to TKA as well as previous hip fusion to THA [12,13]. To our knowledge, this is the first report of this technology being used for revision of a total knee replacement.

Currently, the software is indicated for usage in primary hip and knee arthroplasty only, so its usage in this instance is off-label. This study serves as a proof of concept that the software can be used for this subset of revision surgery. The authors hope that with continued experience with the technique and further advancements of the software, the technology can be used for a wide range of total joint procedures. The authors hypothesize that with better mechanical alignment of the components and better balance of the components, the burden of re-revision surgery could ultimately be decreased and patient satisfaction after revision surgery can be improved, although further studies are needed. In the cases presented here, patients achieved satisfactory outcomes with well-aligned revision TKA components and excellent range of motion and were reportedly pleased with the results of their respective surgeries. Future studies will investigate the comparative functional results and patient satisfaction of standard revision TKA and CT-based robotic-assisted surgery.

This described technique (Fig. 8) offers the surgeon many advantages over traditional instrumentation. The preoperative CT scan affords the surgeon an abundance of information that may better allow preoperative planning. Intraoperatively, the software gives the surgeon much more flexibility to make incremental changes to the component positioning to achieve a balanced knee with symmetric flexion and extension gaps. With real-time feedback of range of motion and ligament balance, the surgeon has more objective data to use to try to optimize patient outcomes, as opposed to surgeon’s experience or tactile feedback. Further studies are needed to evaluate improvements in patient satisfaction and component survival for revision robotic-assisted TKA surgeries.

In the setting of revision surgery, accurate registration presents a significant challenge. The preoperative CT scan must be verified to be of sufficient quality, and in these cases, the authors found several landmarks helpful in accurate registration, including the medial and lateral epicondyles of the femur, the anteromedial metaphyseal region of the tibia, and the tibial tubercle (Fig. 1). This pattern has been reproduced in subsequent surgeries and found to be reliable. Verifying the registration with components removed (Fig. 2) reinforces confidence in the accuracy of the registration. Refinement of the registration process in the revision setting is ongoing, and scrutiny should be used intraoperatively to avoid errors. The accuracy of revision registration should be held to the same standards as in primary surgery, and the green probe may be used to verify concordance between the observed bony landmarks and the corresponding landmarks in the Mako software.

The authors acknowledge that there are several obstacles to widespread adaptation of CT-based robotic-assisted surgery in the revision setting. The robotic-assistance arm software is costly, and further studies looking at whether the cost of obtaining the software is mitigated by the cost for the revision surgery are needed. By diminishing bony resections and need for augments and possibly improving survival of the implants, hopefully the cost burden of revision surgery could be improved. Metaphyseal cones have been shown to be equivalent to longer diaphyseal-engaging stems that require offset [16]. Thus, a smaller and less expensive construct can be used for robotic-assisted revision TKA. A balanced and aligned TKA should only need a regular posterior-stabilized polyethylene component because of proper gap balancing and ligamentous tensioning throughout the knee range of motion. In theory, the need for a constrained polyethylene component should only be used if gross medial-lateral instability is present because of the increased stresses experienced at the tibial tray-cement-bone interface which may contribute to future implant loosening.

In this case report, only 2 patients had sufficient follow-up to warrant inclusion. The authors recognize that they are unable to make conclusions as to whether robotic-assisted revision TKA is superior to conventional revision TKA with jigs based on the limited data available. This technology does also require the use of CT, and there have been concerns raised as to the potential harm associated to the radiation exposure (0.16 mSv for standard knee CT and 4.8 mSv for Makoplasty protocol) as well as added cost [[26], [27], [28]]. Finally, the authors recognize that this technique is an off-label use of the robot-assisted technology. The hope is that with further research and development, this technology can be approved for this indication.

## Summary

The use of robotic-assisted surgery is increasing, and the indications for its use in various surgical techniques are expanding. To our knowledge, this article is the first published demonstration of the use of this technology in revision TKA. Further studies are warranted in examining whether this technology can improve longevity of components or patient-reported outcome measures, and this article serves as a proof of concept that the technology can be used in revision TKA.

## Conflicts of interest

The authors declare that they have no known competing financial interests or personal relationships that could have appeared to influence the work reported in this article.

The authors declare the following financial interests/personal relationships which may be considered as potential competing interests: MWB serves as an Arthroplasty Today Editor. He was not involved in the peer review process for this manuscript. MB also has professional relationships with Smith & Nephew, Osso VR, Stryker, Zimmer/ Biomet, and Depuy.

## Informed patient consent

Complete written informed consent was obtained from the patient for the publication of this study and accompanying images.

## Appendix A Supplementary data

Conflict of Interest Statement for All Authors
