# Supplementary material for: Revision Total Knee Arthroplasty Using Robotic Arm Technology
Source: Arthroplast Today. 2021 Dec 10;13:35–42. doi: 10.1016/j.artd.2021.11.003 (PMC8666608; doi:10.1016/j.artd.2021.11.003)
Supplement: Conflict of Interest Statement for All Authors [file mmc1.pdf]

CONFLICT OF INTEREST STATEMENT

American Association of Hip and Knee Surgeons

(Adopted from the American Academy of Orthopaedic Surgeons disclosure statement)

The following form **must be filled out completely and submitted by each author (example, 6 authors, 6 forms). All items require a response. If there is no relevant disclosure for a given item, enter "*None*."**

Manuscript Title

1. Royalties from a company or supplier (The following conflicts were disclosed)

   None

2. Speakers bureau/paid presentations for a company or supplier (The following conflicts were disclosed)

   None

3A. Paid employee for a company or supplier (The following conflicts were disclosed)

   NO

3B. Paid consultant for a company or supplier (The following conflicts were disclosed)

   No

3C. Unpaid consultants for a company or supplier (The following conflicts were disclosed)

   No

4. Stock or stock options in a company or supplier (The following conflicts were disclosed)

   No

5. Research support from a company or supplier as a Principal Investigator (The following conflicts were disclosed)

   No

6. Other financial or material support from a company or supplier (The following conflicts were disclosed)

   No

7. Royalties, financial or material support from publishers (The following conflicts were disclosed)

   NO

8. Medical/Orthopaedic publications editorial/governing board (The following conflicts were disclosed)

   No

9. Board member/committee appointments for a society (The following conflicts were disclosed)

   No

Each author must sign AND print or type his/her name, date and submit a separate form

In addition, one BLINDED Conflict of Interest form (no author names used) should be submitted per manuscript with all author disclosures.

Micah MacAskill	[signature]	9/8/21
Author Name (Print or Type)	Author Signature	Date

CONFLICT OF INTEREST STATEMENT

American Association of Hip and Knee Surgeons

(Adopted from the American Academy of Orthopaedic Surgeons disclosure statement)

The following form **must be filled out completely and submitted by each author (example, 6 authors, 6 forms). All items require a response. If there is no relevant disclosure for a given item, enter "*None.*"**

Manuscript Title

1. Royalties from a company or supplier (The following conflicts were disclosed)

None

2. Speakers bureau/paid presentations for a company or supplier (The following conflicts were disclosed)

None

3A. Paid employee for a company or supplier (The following conflicts were disclosed)

None

3B. Paid consultant for a company or supplier (The following conflicts were disclosed)

None

3C. Unpaid consultants for a company or supplier (The following conflicts were disclosed)

None

4. Stock or stock options in a company or supplier (The following conflicts were disclosed)

None

5. Research support from a company or supplier as a Principal Investigator (The following conflicts were disclosed)

None

6. Other financial or material support from a company or supplier (The following conflicts were disclosed)

None

7. Royalties, financial or material support from publishers (The following conflicts were disclosed)

None

8. Medical/Orthopaedic publications editorial/governing board (The following conflicts were disclosed)

None

9. Board member/committee appointments for a society (The following conflicts were disclosed)

None

Each author must sign AND print or type his/her name, date and submit a separate form

In addition, one BLINDED Conflict of Interest form (no author names used) should be submitted per manuscript with all author disclosures.

Baylor Blickenstaff, MD	[signature]	9-8-2021
Author Name (Print or Type)	Author Signature	Date

Caughran - Arthroplasty Today

CONFLICT OF INTEREST STATEMENT

American Association of Hip and Knee Surgeons

(Adopted from the American Academy of Orthopaedic Surgeons disclosure statement)

The following form **must be filled out completely and submitted by each author (example, 6 authors, 6 forms). All items require a response. If there is no relevant disclosure for a given item, enter "*None*."**

Manuscript Title

1. Royalties from a company or supplier (The following conflicts were disclosed)

   None

2. Speakers bureau/paid presentations for a company or supplier (The following conflicts were disclosed)

   None

3A. Paid employee for a company or supplier (The following conflicts were disclosed)

   None

3B. Paid consultant for a company or supplier (The following conflicts were disclosed)

   None

3C. Unpaid consultants for a company or supplier (The following conflicts were disclosed)

   None

4. Stock or stock options in a company or supplier (The following conflicts were disclosed)

   None

5. Research support from a company or supplier as a Principal Investigator (The following conflicts were disclosed)

   None

6. Other financial or material support from a company or supplier (The following conflicts were disclosed)

   None

7. Royalties, financial or material support from publishers (The following conflicts were disclosed)

   None

8. Medical/Orthopaedic publications editorial/governing board (The following conflicts were disclosed)

   None

9. Board member/committee appointments for a society (The following conflicts were disclosed)

   None

Each author must sign AND print or type his/her name, date and submit a separate form

In addition, one BLINDED Conflict of Interest form (no author names used) should be submitted per manuscript with all author disclosures.

Alexander T Caughran MD	[signature] MD	9/8/21
Author Name (Print or Type)	Author Signature	Date

CONFLICT OF INTEREST STATEMENT

American Association of Hip and Knee Surgeons

(Adopted from the American Academy of Orthopaedic Surgeons disclosure statement)

The following form **must be filled out completely and submitted by each author (example, 6 authors, 6 forms). All items require a response. If there is no relevant disclosure for a given item, enter "*None.*"**

Manuscript Title

1. Royalties from a company or supplier (The following conflicts were disclosed)
   None

2. Speakers bureau/paid presentations for a company or supplier (The following conflicts were disclosed)
   Smith & Nephew

3A. Paid employee for a company or supplier (The following conflicts were disclosed)
   None

3B. Paid consultant for a company or supplier (The following conflicts were disclosed)
   Smith & Nephew

3C. Unpaid consultants for a company or supplier (The following conflicts were disclosed)
   Osso VR

4. Stock or stock options in a company or supplier (The following conflicts were disclosed)
   Stryker

5. Research support from a company or supplier as a Principal Investigator (The following conflicts were disclosed)
   None

6. Other financial or material support from a company or supplier (The following conflicts were disclosed)
   Educational Support: Stryker – Smith & Nephew – Zimmer/Biomet - Depuy

7. Royalties, financial or material support from publishers (The following conflicts were disclosed)
   None

8. Medical/Orthopaedic publications editorial/governing board (The following conflicts were disclosed)
   Editorial Board Arthroplasty Today

9. Board member/committee appointments for a society (The following conflicts were disclosed)
   AAHKS Patient Education Committee; West Virginia Orthopaedic Society Education Committee

Each author must sign AND print or type his/her name, date and submit a separate form

In addition, one BLINDED Conflict of Interest form (no author names used) should be submitted per manuscript with all author disclosures.

Matthew Bullock DO	(Bullock)	9/9/21
Author Name (Print or Type)	Author Signature	Date
